# Commentary: The Sequence and Reconstructive Modality of Breast Cancer Treatments Affects Wait Times to Adjunctive Therapies in Patients Undergoing Mastectomy with Immediate Breast Reconstruction

**DOI:** 10.1177/22925503251330644

**Published:** 2025-03-28

**Authors:** Mitchell H. Brown

**Affiliations:** 1 Plastic and Reconstructive Surgeon and a Professor in the Division of Plastic Reconstructive and Aesthetic Surgery, Department of Surgery, 7938 University of Toronto, Toronto, Canada

The intersection of ablative cancer surgery and reconstructive surgery present both opportunities as well as challenges. A primary tenet of medical care is that first, *Do No Harm*. To state the obvious, cancer care is about curing disease and prolonging life, while reconstructive surgery addresses issues of living well and maintaining or maximizing quality of life. It is imperative that decisions related to reconstructive surgery, adequately respect the importance of cancer care and that all reasonable steps are taken to minimize any negative impact on the delivery of treatment of the disease. The authors of the paper, “The Sequence and Reconstructive Modality of Breast Cancer Treatments Affects Wait Times to Adjunctive Therapies in Patients Undergoing Mastectomy with Immediate Reconstruction,”^1^ should be congratulated for their important work looking directly at this issue.

The authors present a retrospective review of 337 patients post-therapeutic mastectomy and immediate breast reconstruction which was either purely alloplastic or autologous. Patients were divided into a surgery first group (SF) and a neoadjuvant chemotherapy first group (NC). The authors looked at wait times between care pathway milestones and compared these to national standards, using the 2019 Pan Canadian Breast Cancer Surgery Standards^2^. Not surprising, SF patients had longer wait times from consult to treatment initiation (25 days) and from first to second treatment modality (23 days) when compared to NC patients. Only 29% of SF patients met standards of receiving treatment within 4 weeks of consult. These delays are likely related to the added time necessary to incorporate a reconstructive surgery consultation, coordination for combined surgery, preparation for surgery and adequate surgical recovery to allow for the institution of second modality care.

Method of reconstruction had an impact on wait times. In the SF group, surgery occurred faster in patients undergoing alloplastic reconstruction. This was most certainly due to factors such as shorter required operating room time, and fewer necessary hospital resources including inpatient beds and step-down units. Some institutions have tried to address this issue by offering tissue expander insertion as an initial procedure in all patients desiring reconstruction. Expanders can then be replaced with implants or autologous tissue, or in some cases, simply removed altogether. Conversely, alloplastic patients were less likely to meet the 28-day target for initiation of radiation following surgery than the autologous group. This was noted in both the SF and NC cohorts and likely reflects the importance of having a stable soft tissue environment surrounding a device prior to radiation. As stated by the authors, this may also be due to the fact that patients known in advance to be likely to receive radiation are less inclined to be reconstructed with implants.

Complications were not significantly different in the SF and the NC groups. This implies that if patients wait the recommended time after chemotherapy before undergoing surgery, they will have a similar risk profile to those that have not had chemotherapy.

Given the retrospective nature of this study, there are a few limitations worth noting. There was a trend to more younger patients in the NC group. This cohort also had a notably higher percentage of patients with invasive disease. It is possible that this impacted perceived urgency for institution of therapy. The authors also make the important point that surgical delays may not be entirely due to reconstruction. Impact of mastectomy-related complications would require a comparison group that chooses mastectomy alone without reconstruction.

This excellent paper highlights the need for a comprehensive multidisciplinary approach to the management of the breast cancer patient. This requires a commitment from all involved clinicians to prioritize scheduling and availability. It also requires the support of hospitals to provide adequate resources in terms of staffing, funding, flexible operative time, state-of-the-art devices and materials, and support for research, education and community outreach. Breast reconstruction is constantly evolving. Techniques such as limited muscle harvest, pre-pectoral implant placement, direct-to-implant reconstruction, and expanded use of nipple-sparing mastectomies are allowing surgeons to deliver outcomes that were previously considered unattainable. In this patient population, complications come with a steep cost. This paper is an important reminder to prioritize safety and the delivery of care in patients being treated for breast cancer.
